# Applying Transdisciplinary Thinking to Pastoral Livelihoods and Environments

**DOI:** 10.3390/ani15131933

**Published:** 2025-06-30

**Authors:** Keith Woodford, Xiaomeng Lucock, Derrick Moot

**Affiliations:** 1 Department of Land Management and Systems, Faculty of Agribusiness and Commerce, Lincoln University, Lincoln 7647, New Zealand; kbwoodford@gmail.com; 2 Department of Agribusiness and Markets, Faculty of Agribusiness and Commerce, Lincoln University, Lincoln 7647, New Zealand; 3 Department of Agricultural Sciences, Faculty of Agriculture & Life Sciences, Lincoln University, Lincoln 7647, New Zealand; derrick.moot@lincoln.ac.nz

**Keywords:** pastoral systems, transdisciplinary thinking, system thinking

## Abstract

Transdisciplinary thinking lies at one end of a continuum within system thinking, with discipline-based approaches at the other end. Interdisciplinarity and multidisciplinarity are intermediate domains within this continuum. Transdisciplinary thinking is unique in always starting with problem-structuring related to contexts, in which people, typically with multiple and competing objectives, interact with a biophysical world. As such, transdisciplinary thinking is particularly relevant to pastoral systems where livelihoods and environmental issues intersect, and where multiple stakeholders are the norm. Integration, both within transdisciplinary thinking and consequent action, is particularly challenging. This is because there is no quantitative methodology that can capture the complex essence of transdisciplinary issues that encompass both human and biophysical disciplines. Nevertheless, a transdisciplinary approach provides a framework for civilised debate and communication within a broad framework of policy generation. We illustrate these issues with two highly contrasting studies, these being pastoralism at the country level in New Zealand and at the county level in Qinghai on the Qinghai-Tibet Plateau in Western China. Both case studies are characterised by complex property rights within a dynamic resource-constrained environment, in which environmental issues have planetary implications that extend well beyond the bounds of the pastoral system itself.

## Simple Summary

Pastoral farming is under intense global scrutiny from societal and environmental perspectives. We provide two contrasting case studies, these being New Zealand pastoralism at the country level and pastoralism at the county level in Qinghai on the Qinghai-Tibet Plateau in Western China, to illustrate the potential role of transdisciplinary thinking in structuring these complex problems. The starting point of any transdisciplinary study is to define the biophysical context, together with identification of all stakeholder groups. There is explicit recognition that conflicting objectives may be a consequence of different value systems. Investigators should assess their own value systems through a process of reflexivity to avoid unconscious bias. Transdisciplinary studies contrast with disciplinary studies in that the context always precedes the choice of disciplines. Given the focus on context, transdisciplinary studies are always applied with choice and integration of disciplines as a consequence of the specific transdisciplinary context. Unfortunately, transdisciplinary thinking does not always lead to transdisciplinary actions. This is because many pastoral problems are inherently ‘wicked problems’, where conflicting objectives prevent non-conflicting outcomes. However, a transdisciplinary approach, at the very least, provides a framework for civilised debate and communication within a broader framework of policy generation.

## 1. Introduction

The role of pastoral farming is under intense global scrutiny from societal and environmental perspectives. Many disciplines have been, and are being, drawn upon by investigators working from within each discipline to identity a range of important issues, with this leading to potential changes to pastoral systems that could and perhaps should be made. However, each issue is typically investigated within a reductionist framework, with the investigatory process starting from within the defined discipline in which the researchers operate, and with results typically measured in relation to a single defined objective.

This situation reflects that over the last 150 years and arguably much longer, as scientific knowledge has grown, most of this knowledge has developed within a culture of specialisation. The standard way that scientists achieve both success and acknowledgement has been by seeking out new insights relating to more and more specialised fields. Universities and research organisations are also typically structured on the basis of disciplinary specialisation.

There is no doubt that disciplinary science has been highly effective in organising science within an internally coherent framework that has worked particularly well in a world of expanding resource availability, particularly synthetic fertilisers, to meet a growing global population. This occurred in association with a societal consensus that economic growth was a key measure of societal wellbeing, and even diversification into activities such as agritourism is still largely aimed at generating economic benefits [1]. However, society now faces major issues related to resources that are increasingly constrained and where the pathways forward are less clear. This emergent situation can be characterised as comprising a set of ‘wicked problems’ [2], which have contested objectives and have no clear pathway to an uncontested solution. Objectives can be contradictory, with technical solutions in relation to one objective having either negative outcomes or large opportunity costs in relation to other objectives. This is where modern-day pastoral systems sit, as complex human activity systems employing both extensive and intensive animal husbandry systems at different levels of land development and capital intensity, plus environmental externalities that lie outside the commonly accepted boundaries of the farming system.

Facing such a complex ‘crossroad’, we explore and present an overarching transdisciplinary framework that can assist in the integration of contributions from potentially many disciplines in relation to pastoral livelihoods and pastoral environments. Key attributes of these pastoral livelihoods and environments are objectives that are multiple, at times conflicting, and influenced by a range of value systems. In essence, this paper is a thought piece, drawing on both theoretical literature and our own experiences in specific situations, to push back the boundaries of transdisciplinary pastoral system analyses.

Our reason for writing this paper is that we believe that transdisciplinary thinking needs to have an increased role in structuring complex policy issues relevant to pastoral livelihoods and associated environmental issues. We also note here at the outset that some of the thinking and research described in the academic and professional literature as being ‘transdisciplinary’ is a loose use of the term. Here, we emphasise that transdisciplinary thinking is different from multidisciplinary and interdisciplinary thinking. They are complementary terms, but they are not synonymous.

Our hope is that by focusing on fundamental tenets of transdisciplinary thinking, we can provide a framework that facilitates coherent debate about alternative pathways that lie ahead for pastoral systems, together with the implications thereof. We contend that the philosophies of transdisciplinary systems as a way of thinking can be applied widely, but that specific methods and solutions are dependent on context. Hence, specific methods and system improvements can only flow from specific and appropriate situation analyses of resources, constraints, objectives, and value systems, together with system interactions and feedback loops.

## 2. Definitions

Before proceeding, it is necessary to define what we mean by a range of terms. We first explain our interpretation of pastoral livelihoods and pastoral environments within a systems framework. We then define our use of the term ‘transdisciplinary thinking’, together with the meaning of integration and context as used within a transdisciplinary philosophy. In doing so, we acknowledge that others may define these terms somewhat differently; however, without clarity as to how we are using these terms, there can be no clarity in what follows.

### 2.1. Pastoral Livelihoods

We define pastoral livelihoods as all livelihoods where pasture-eating animals play a major part in human livelihood. In practice, this means we are thinking of all ruminant species, primarily sheep (*Ovis aries*), cattle (*Bos taurus*), and goats (*Capra hircus*), but also other species, such as yaks (*Bos grunniens*), camelids (*Camelidae*), and deer (*Cervidae*), which are tended to in pastoral environments. We are inclusive of grazing and browsing species. We are also inclusive of a continuum from extensive rangelands to irrigated pastures and recognise that supplements purchased from outside the farm boundary can be an integral part of that system. We acknowledge that pastoral systems are typically embedded within value chains that extend back from consumers to processors to producers to input suppliers, and that multiple stakeholders lie within those value chains. We also acknowledge that pastoral externalities, in particular but not confined to greenhouse gas emissions, water pollution, nutrient loss, and erosion, create stakeholders outside the direct pastoral value chain.

### 2.2. Pastoral Environments

We define pastoral environments as having both biophysical and social dimensions, with those extending well beyond the physical boundaries of farms and rangelands. Soil fertility, erosion, nutrient loss, downstream pollution, greenhouse gases, off-farm stakeholders, governmental institutions, regulations, research and development structures, cultural factors, and faith-based ideals are all relevant.

### 2.3. Transdisciplinary Thinking

A fundamental principle of transdisciplinary thinking is that no discipline or value system should be given precedence at the outset of an investigation. Rather, the relevant disciplines and stakeholder objectives are emergent from a situation analysis of the issues and problems that are to be investigated. Similarly, it is a flawed approach to assume a single objective at the outset. Giving precedence to a single objective at the outset is almost certain to preclude key stakeholders and thereby miss important causal links within the system. It is the comprehensive identification of the system under consideration and its boundaries that has to come first. This also includes identification of externalities and flows across the system boundary.

In defining transdisciplinary thinking, it can be helpful to think of disciplinary and transdisciplinary thinking as opposite ends of a philosophical continuum [3]. The intermediate domains within the continuum are multidisciplinary and interdisciplinary thinking. We emphasise that multidisciplinary, interdisciplinary, and transdisciplinary thinking are companion terms, but they are not synonyms. There is scope for overlap between the boundaries of these specific domains, but failure to appreciate the fundamental differences between multi-, inter-, and trans- has, at times, led to a lot of confusion [4].

Here, and in relation to this philosophical continuum, we define multidisciplinarity as research and teaching situations where disciplines operate in parallel in investigating and interpreting a system, but where each discipline operates from within its own discipline. For example, a multidisciplinary study of a pastoral system might include soil, plant, and animal scientists. Each will study the pastoral system from their own perspective and each will identify weaknesses that can be addressed by their discipline. A joint report will likely have defined and separate domains relating to soils, pastures, and animals, including the disciplinary language of each.

**Figure 1:**
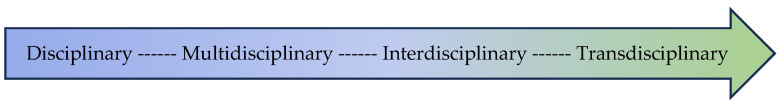
Philosophical continuum from disciplinary to transdisciplinary thinking.

In contrast, an interdisciplinary study of the same pastoral system would include explicit interactions between the disciplines, typically including at least all of soils, plants, and animals, and how each affects the other. Hence, any report based on interdisciplinarity will have explicit consideration of flows between the organisational domains of soil, plants, and animals, and recognise that there will be nutrient and other flows in both directions. The study may start with a static situation analysis at a particular time, but the dynamics of the stocks and flows of variables over time, including feedback loops, are likely to be key to understanding how the system operates, and how the overarching system might be changed. We illustrate this philosophical continuum below in Figure 1.

In moving further along the philosophical continuum to transdisciplinary thinking, a study is unlikely to be comprehensive unless the system is framed as a human activity system that involves both the hard sciences where experimentation is the norm, and the soft sciences of economics, sociology, and management, where controlled experimentation with treatments and controls is seldom feasible. There are likely to be multiple stakeholders within the transdisciplinary system and these stakeholders are likely to have competing objectives, often underpinned by different fundamental value systems, and are likely to be impacted both by institutions and associated power structures.

### 2.4. Integration

In moving along the continuum from disciplinary thinking to transdisciplinary thinking, the key stages within that continuum can be described in terms of the level of integration and the ease thereof. Here we define integration in broad terms as a catch-all phrase, by which we mean the process whereby individual disciplines are brought together.

Within disciplinary science, any integration that occurs will be within a discipline and is likely to relate to changes to an individual output variable compared with changes in an input variable, with the standard scientific method involving one or more treatment levels in relation to a constant-level control. A more sophisticated experimental design might include multiple levels of two input variables and variable interactions. Disciplinary studies are typically amenable to either or both mathematical calculus and biostatistics.

The above situation with quantitative analyses also typically applies within multidisciplinary studies, but with quantification occurring within single disciplines, using either or both calculus and biostatistics. Any integration between disciplines is likely to be qualitative, non-numerical, and text-based, and occur towards the end of any report. We recognise here that modelling, which encompasses many varied approaches, can combine both quantitative and qualitative data at times. However, for the models to function and serve the purposes of desired predictability, there are normally strict assumptions made to restrict the number of variables to control the level of uncertainty within the models, which often makes it distant from the complexity of real-world problems.

Nevertheless, within interdisciplinary studies, where there is explicit interaction between disciplines, the use of either calculus and biostatistics is challenging and often infeasible, except when looking at individual components of the analysis as parts of a greater whole. However, the use of causal loops to identify interactions between disciplinary domains, extending through to the potential use of system dynamics modelling [5,6], can greatly assist with development of an interdisciplinary process.

With transdisciplinary studies, we cannot identify any existing study that has been analysed at the overarching holistic level using either mathematical calculus or biostatistics. This is because such studies are, by definition, driven and constrained by physical, biological, and institutional parameters, together with culturally based value systems. Hence, there is no universal calculus to guide the integration process. There is also no single method or system of reasoning. However, there are systems of logical exposition and possible trade-offs between objectives that can be made explicit, and trade-offs may well be quantifiable. We see the explicit recognition of multiple objectives within a complex sociological context associated with multiple stakeholders, institutional structures, and power structures as being the key distinction between interdisciplinary and transdisciplinary thinking.

## 3. Contexts and Methods

In this paper, we focus primarily on two contexts as case-study applications of transdisciplinary thinking. One of these is temperate New Zealand, where all three authors currently reside. The second context is a high-altitude, low-rainfall region within the Qinghai Province of Western China, where two of us have worked extensively. We could have used additional contexts, having collectively worked in and studied in pastoral environments in more than 20 countries. However, for simplicity we have focused primarily on New Zealand and Western China, in large part because they provide such stark contextual environments. We also acknowledge here as authors of this paper that our own integrative philosophies and perspectives presented here have been influenced by experiences in many other contexts.

### 3.1. The Importance of Context

Transdisciplinary thinking and the associated integration can be described in terms of a high-level philosophy as set out above, but the application is always context-specific. Accordingly, if progress is to be made in real-life transdisciplinary situations, then understanding the context must precede the choice of appropriate disciplines. By context we mean the combination of physical, biological, socio-economic, and institutional parameters of the system, together with stakeholder objectives and value systems. Context can also be thought of in terms of the historical, current, and future systems, and the dynamics by which change has occurred and/or may occur in future.

The context can also be defined at multiple levels, sometimes within a hierarchy. For example, pastoral systems operate at a global level with global implications. They also operate at country, regional, and farm levels, as determined by specific biotic, abiotic, edaphic, and topographical factors, together with a range of current and historical socio-economic factors. These factors lie both within and external to the assumed system boundary in influencing current and future livelihoods. Transdisciplinary system studies may also take place at the level of industries or firms from the perspective of operatives working from within the system, albeit influenced by major external factors outside the system boundary that may still need to be identified.

The importance that we are placing on context and its placement at the forefront of applying transdisciplinary thinking can be illustrated by a comparison with the norms of disciplinary science, where it is normal for scientific techniques that have been built on known scientific principles to take a lead role. Scientists then look for applications in which the principles and techniques associated with their specialisation can be tested and applied, leading to further extension of knowledge. In contrast, within a transdisciplinary framework, it is the context and the associated identified issues that determine the relevant disciplines, objectives, and prior knowledge, as well as the breadth of value systems. This process starts with a situation analysis defining the system. A consequence of the placement of context at the forefront is that most transdisciplinary studies are applied studies.

In advocating for the transdisciplinary approach, we are not arguing against disciplinary approaches within research and education. Nor are we in any way arguing against multidisciplinary and interdisciplinary investigations. The evidence for the role of disciplinary research is incontrovertible [7]. However, when it comes to applied investigations and, importantly, policy development, that is when we advocate that the transdisciplinary approach has an important role to play.

At this point, we also choose to make a clear distinction between transdisciplinary thinking and subsequent transdisciplinary actions to modify the system. By transdisciplinary thinking, we mean the process of seeking knowledge about a human activity system within a comprehensive multifactorial framework of context and analysis, thereby contributing to knowledge and understanding about the system. This includes both within-system interactions and flows of material and information across the system boundary. This is a powerful framework for analysis of complex situations, but when it comes to consequential actions there may be major institutional barriers and unresolved conflicts that either inhibit or preclude stakeholder actions from occurring within a participatory framework. We will return to the challenges of transdisciplinary actions later in this paper in relation to our case studies. Initially, our focus is on analysing and understanding situations and contexts within a transdisciplinary setting as a necessary prelude to actions.

### 3.2. Development of Systems and Transdisciplinary Thinking Within Agriculture and Pastoral Systems

Before moving to the specific contexts of the transdisciplinary systems thinking approach to pastoral farming, it is appropriate to consider something of the history of systems thinking and its historical applications within agriculture. We also choose to say something about our own exposures to system thinking. We do this because systems thinking is indeed a philosophy, which implies that, inevitably, there are elements of subjectivity therein, influenced by experiential shaping forces. Acknowledging and coming to terms with author subjectivity through an explicative process of reflexivity is a fundamental element in the communication of transdisciplinary thinking.

In researching for this paper, we quickly came to the conclusion that there is no clear chronology in regard to the development of general systems thinking nor its application to agriculture, nor to the subsequent journey from that initial systems thinking through to transdisciplinary thinking. Rather, systems thinking in both agriculture and other fields of human endeavour emerged over time from within individual disciplines. These were led by people who saw the need to work across disciplines if they were to address real-world problems. Hence, there is no single foundational root or stem to agricultural systems thinking. Indeed, the development of systems thinking within agriculture could be described by what Ackoff said back in 1974 [8] and, talking in general terms about human activity systems, called a ‘mess’, with different people and groups coming together over time but in a chaotic way. This has impacted on how specific terminology is often used in different ways by different groups.

An early integrative thread between biological systems and farm economics was Dent and Blackie’s ‘System Simulation in Agriculture’, published in 1979 [9]. Over time, systems thinking developed its own structures and norms, with so-called ‘hard systems’ focusing on the biological sciences and systems modelling, typically with a productivity-based objective, and with another thread focusing on ‘soft systems’ [10,11,12], with an emphasis on people, organisations, and cognition. A further thread explored the use of systems thinking within agricultural education as a philosophy that can underpin the structure of learning within institutions [13,14]. The Elsevier journal Agricultural Systems commenced publication in 1976 [15] and provided a home for both hard and soft systems papers, but there is limited evidence of bringing those threads together. Using the definitions of our own paper here, most but not all of the research reported within the journal Agricultural Systems has been interdisciplinary, with a predominant focus on either the hard or soft disciplines and limited integration between the two.

In New Zealand, a farmer-centric farm systems analysis was fundamental to the way farm management was taught at Lincoln University, which is our own alma mater from prior to the Second World War. This farmer-centric farm systems analysis involves the integration of both hard and soft sciences in relation to farmer objectives. It could be described as systems thinking without the systems terminology. This was in contrast to the typical situation elsewhere, with farm management structured as a subset of agricultural economics and the biological sciences siloed within their disciplines. For many years, all students undertaking the agricultural science degree at Lincoln University (at that time Lincoln College of University of Canterbury) were required to take courses in both agricultural economics and system-focused farm management, with the latter supported by 48 weeks of practical farmwork and multidisciplinary field trips that were underpinned by the “hard sciences”. This farmer-centric approach then evolved into postgraduate and research activities within an integrated bio-economic systems framework at both Lincoln University and New Zealand’s Massey University, albeit with the farm boundary as the system boundary. This included pastoral system optimisation procedures within linear programming and often incorporated risk [16] and pastoral-system simulation modelling [17,18]. Specific farmer-first studies then developed as a formal sub-thread of farming systems at Massey University [19]. This identified that pastoral farmers had diverse objectives and were typically rational in pursuing those objectives, although often constrained by resources [20]. This research occurred in parallel with farmer-first studies in developing countries, with leadership from Sussex University focusing on the importance of participatory processes [21,22,23].

From the early 1990s, there was an increasing focus globally within agricultural systems on agro-ecological systems, which highlighted the likelihood of competing objectives and moved systems thinking towards so-called ‘wicked problems’ with no obvious solution. Within soft systems, there was an increasing focus on learning and cognition, recognising the importance of reflexivity and reflective learning whereby system researchers conducted self-analysis of their own perspectives, assumptions, and otherwise non-disclosed value systems [24].

Our own interrogation of Google Scholar using the terms ‘multidisciplinary’, ‘interdisciplinary’, ‘transdisciplinary’, ‘agriculture’, and ‘pastoral systems’ in various combinations and over different chronologies supports the premise that the use of the term ‘multidisciplinary’ is in decline, whereas ‘interdisciplinary’ and ‘transdisciplinary’ are terms enjoying increasing popularity. We voice this as a premise that requires further documentation, but in which we have considerable confidence. However, further interrogation of databases tends to confirm that the current situation is one where many authors loosely describe their land and livelihood work as ‘transdisciplinary’, but for which we would describe ‘interdisciplinary’ or even ‘multidisciplinary’ as being more appropriate. Also, the projects that have attempted to use transdisciplinarity within an action-research framework have often struggled with a fundamental reality that the issues at stake remain part of a wicked problem with contested objectives, resource constraints, institutional constraints, and power relationships.

In regard to pastoral situations, the recent transdisciplinary literature relates primarily to rangeland situations and is led by a focus on livelihoods. It is typically participatory, at least to the extent of engaging with pastoralists; however, action research that has a transdisciplinary focus remains problematic and challenging [25]. Much pastoral research remains embedded within individual disciplines and published in disciplinary journals. We will illustrate this reality within our case studies.

One of the early contributors to transdisciplinary thinking was Ray Ison [12], who emphasised the importance of reflexivity leading to ongoing testing of one’s own assumptions and world view. This issue of reflexivity is fundamental to the process of reducing the unconscious impact of personal biases and personal value systems.

It is in relation to reflexivity and world view, and the importance thereof, that we add something about our own background as it relates to this paper. One of us has a China-based education and was first exposed to systems thinking as a postgraduate at Lincoln University. The other two of us are Bachelor of Agricultural Science graduates from Lincoln University, with one of us specialising thereafter in farm management and livelihood issues, and the other specialising in applied agronomy, reflected in his email descriptor that ‘excellent agricultural science only happens in the field’. All of us teach and research systems frameworks, with a focus on linkages and big picture thinking’, but also drilling down from that at times, as a matter of academic survival, into disciplinary issues that feed back to the bigger picture. All of us regard farmer interactions as where we do much of our own learning.

## 4. Results

### 4.1. Applying Transdisciplinary Thinking to New Zealand Pastoral Systems

#### 4.1.1. Contextual Background of New Zealand

New Zealand was first settled in approximately 1300 CE by Polynesian seafarers. These first settlers lived mainly off coastal fishing and the growing of some vegetables, mainly sweet potatoes. There were no ruminant animals. European settlers started arriving around 1800 CE, associated initially with whaling activities, but by 1840 developing settlements and pastoral farming based largely on English farming methods [26]. Fast forward almost over 180 years with continued migration from other nations around the world, New Zealand is now a nation of approximately 5.0 million people, comprising European (68%), Māori (18%), Asian (17%), Pacific Island (9%), and other ethnicities (3%) [27].

New Zealand is geographically a South Pacific nation of approximately 26.8 million hectares (including lakes), with a maritime temperate climate and prevailing westerly weather systems. The topography is mountainous and hilly. Approximately 8 million hectares have a protected classification as national parks and or other conservation classifications. The remaining land is predominantly used for pastoral farming and production forestry using introduced species (mainly *Pinus radiata* from the USA), plus some arable agriculture, horticulture, and wine.

Prior to human habitation, the land cover was predominantly scrub and forest. Soils in their natural state were deficient in phosphorus and sulphur. As pastoral farming developed since the 19th century, specific locations also required inputs of cobalt and selenium to achieve ruminant health.

Approximately 80% of New Zealand’s export income is from physical products derived from primary industries. Pastoral-sourced products comprise ~50% of these physical exports [28]. In contrast, New Zealand has limited manufacturing industries and lacks scale to be internationally competitive in most manufactured goods. It is the primary industry exports that pay for most imports of manufactured goods.

In New Zealand, there are approximately 10,500 dairy herds on the easier country, averaging ~450 milking cows plus young stock per herd [29]. Most farms range between 200 and 1000 cows and are family owned with non-family labour also employed. Some farmers own multiple farms, sometimes in joint ventures with other farmers, and these businesses typically employ professional managers within a framework that can be described as ‘family corporates’. Most dairy farms calve all of their cows in spring to align with pastoral growth cycles. This concentrated calving leads to both seasonality and a low cost of production. Any cow that fails to maintain this spring calving is culled for meat, with this being driven by technical and financial efficiency criteria. Milk is chilled on-farm, collected daily by tanker, and taken to large processing factories, often more than 100 km distant. Approximately 80% of dairy farmers belong to the Fonterra mega co-operative, which processes milk on their behalf into multiple products, such as milk powder, butter, and cheese.

The sheep and beef industries comprise approximately 9000 farms of commercial size and provide full-time employment for at least one person. In addition, there are another 13,000 lifestyle properties containing some animals but with the owners working in towns and cities in a range of trades, primary industry processing businesses, and various service industries [30,31]. Commercial-scale sheep and beef farms are typically family-owned, with between one and five working staff including the family owners. A typical sheep and beef farm would comprise between 1500 and 4000 breeding female sheep, plus several hundred beef animals. These can include surplus young stock from the dairy industry. Sheep and beef are widely considered to be complementary pastoral species, with this complementarity linked to different grazing habits of sheep and cattle and also to control of internal parasites. Meat processing is dominated by one farmer owned co-operative, one hybrid co-operative with 50% farmer ownership and 50% Chinese ownership, one privately owned family business and one 100% Japanese-owned business. There are also many smaller meat-processing businesses. Approximately 85% of meat production is exported, mainly in frozen form but with some in chilled form.

New Zealand has seen an ongoing land-use change from sheep to dairy, at varying rates, for approximately four decades, with this driven primarily by price relativities. This land-use change has also led to intensification of input use, including fertiliser and irrigation [32]. However, further conversion of land to dairying from either other pastoral uses or forestry has essentially been precluded by regulation as of 2020 [33].

All New Zealand pastoral industries can be considered as being technically and economically efficient, research-informed industries, with farm owners and managers who are in general well educated, and with many of the younger generation of farmers with a tertiary-level qualification. In recent decades, on-farm production strategies have focused first on optimising, within economic constraints, the production of metabolisable energy from herbage, using fertilisers as determined by soil test results historically for phosphorus and sulphur to support legume growth for nitrogen fixation. On dairy farms, there has been a major shift, which commenced in the late 1980s from clover as the predominant source of nitrogen to the use of inorganic nitrogen fertilisers. However, on sheep and beef farms, the economics of input and output relationships linking nitrogen sources to herbage production has determined that nitrogen-fixing legume species remain the predominant fundamental source of nitrogen for the system [32].

Alongside the production of herbage, there are key foci on maximising both feed utilisation and feed quality, with rotational grazing combined with pasture spelling to allow post-grazing regrowth. Given the seasonality of herbage production, seasonal reproduction and slaughter strategies are also fundamental. This seasonality leads to low utilisation of processing facilities when measured on an overall annual basis. Together with long transport distance to the major markets in the Northern Hemisphere, a focus on long-life products, such as milk powders and frozen meat, have been a key feature of New Zealand’s pastoral sectors.

Inevitably, there are tensions between a seasonal production focus and a market-led focus, with consumer demand tending to be non-seasonal. More information on the technical and value chain aspects of these New Zealand pastoral systems is provided by [34,35], with both of these papers being part of this special issue of the journal Animals.

There is widespread acceptance but not necessarily unanimity that New Zealand’s pastoral industries, particularly dairy, have reached land-based environmental limits. Total pastoral stock units of sheep, beef cattle, and dairy cattle, with one stock unit being the equivalent of a breeding female sheep, declined 11% between 2012 and 2022 [30]. Much of this decline is linked to land-use changes from pastoral farming to forestry incentivised by carbon credit schemes, with this particularly impacting sheep numbers.

#### 4.1.2. Stakeholder Perspectives on New Zealand Pastoral Systems

We now shift the focus to the diversity of stakeholder perspectives and associated challenges as to how these industries might adapt within a contested environment that would benefit from greater transdisciplinarity in the determination of policy.

In identifying the relevant stakeholders, there are two key realities that have to be acknowledged. The first is that primary industries and pastoral farming in particular underpin the New Zealand economy. They have a dominant role in national exports as documented earlier in this paper, together with the associated fact that primary industries are where New Zealand holds both international competitive and comparative advantages. The second is the reality that there are large environmental externalities beyond the direct supply and value chains that link production and consumer markets, and which impact New Zealand society in general. These environmental impacts include land erosion and associated sedimentation and surface runoff with phosphorus attached, nitrogen leaching into waterways, methane emissions from ruminant digestion, and nitrous oxide emissions from within the system. Accordingly, it is reasonable to state that all members of New Zealand’s society are relevant stakeholders in investigations as to the current role played by agricultural systems as bio-socio-economic systems and how this role could or should change in future.

From a societal perspective, two particularly contentious issues relate to water pollution from pastoral animal excreta and methane emissions from ruminant metabolism. These play out as government-imposed regulations that limit the intensity of pastoral farming. A third issue that impacts both of the above two issues is land-use change from pastoral farming to forestry, with payments for carbon sequestration being a major driver thereof that changes the previously market-led decision making on appropriate land use.

**Water pollution.** The three major pollutants of concern are nitrate leaching, erosion of soils with phosphorus attached, and *E. coli* contamination. In New Zealand, all pastoral farms are required to exclude pastoral animals from streams that have a bank-to-bank width of more than one metre at any point within a property, and with a fencing setback of at least three metres from stream banks [36]. Animals must also be excluded from lakes, natural wetland, and permanent waterways. Slope restrictions also exist to prevent either cash cropping or forage cropping where there is significant potential for soil loss. Restrictions on nitrogen-based fertilisers, limited since 2021 to 190 kg of elemental nitrogen per hectare per annum [37], have the effect of constraining livestock stocking rates, which reduces nitrate leaching and *E. coli* pollution of waterways. Water pollution stakeholders include urban citizens who rely on water sourced from pastoral catchments, plus special interest groups, such as Fish and Game New Zealand, who have significant political influence.

**Methane.** There is considerable societal debate about the importance of ruminant-sourced methane. The fundamental science relating to methane production by ruminant animals as an evolutionary strategy for dealing with the excess of hydrogen from the metabolism of pastoral diets, is well understood, at least by scientists. Similarly, there is widespread acknowledgement by all informed parties that methane is a greenhouse gas. However, there is fervent debate as to how ruminant-sourced methane should be measured relative to carbon dioxide in terms of carbon dioxide equivalence, with specific metrics dependant on judgements as to the appropriate analytical timelines for comparison. The importance of this value-based judgement arises from the atmospheric lifetime of methane being much less than the atmospheric lifetime of carbon dioxide and the contention that methane from ruminants is part of a natural cycle compared with the transformation of fossil carbon to atmospheric carbon that occurs in the energy sector. The challenge of ruminant-sourced methane from pasture-based systems is exacerbated by existing science-based methane reduction strategies using feed additives such as 3-NOP (trade-named as Bovaer^®^, DSM-Firmenich, Amsterdam, The Netherlands) [38], which are limited to formulated feed strategies to which the additives can be readily mixed.

**Land-use change.** There has been widespread conversion of New Zealand hill country pasture to forestry over a 30-year period. This has been driven by the relative economics of hill country pastoralism versus the economics of *Pinus radiata* forestry, with this species originally introduced to New Zealand in the mid-19th Century. Experience soon demonstrated that ‘radiata pine’, as it is known, was much faster growing than native species of trees and, following the Second World War, it became the dominant species within planted forests.

During the 1990s, there was a major pulse of land conversion from sheep to forestry driven by the relative economics of forest products, such as pulp and paper, versus the sheep products of meat and wool. More recently, and particularly from around 2018 through to 2024, the economics of forestry has been driven by the combination of payments for carbon sequestration and a high average price of logs exported for timber to China.

Large-scale conversion of hill country pastoral farms to forestry is opposed by Beef + Lamb NZ, which is the major sheep and beef producer organisation with an industry mandate that includes research and development, plus political lobbying on relevant policies. Beef + Lamb NZ is quick to correctly point out that production forestry excluding any carbon sequestration payments is very much a long-term investment and earns nothing for approximately 25 years [39]. Also, carbon sequestration receipts are a transfer payment within New Zealand’s internal economy rather than an export earner. However, many hill country sheep and beef farmers also recognise that forestry options have been underpinning land values. This is of particular importance to older farmers considering retirement and the sale of their farm to fund that retirement, together with eventually providing a legacy for children who do not wish to be farmers.

Land-use change to radiata pine is also opposed by significant groups in the broader society. Much of this opposition is based on a value-based preference for native rather than introduced forests, but not necessarily recognising that native species are both much slower growing than introduced species and are also particularly susceptible during the establishment phase to introduced pests, such as deer, pigs, goats, and Australian-origin possums (these species were brought to New Zealand by European settlers and subsequently left in the wild, posing a threat to both farm production and native flora and fauna). Mature forests also become steady states in terms of carbon sequestration. This contrasts the cyclic cutting and replanting of radiata pine that provides ongoing sequestration as long as the harvested wood has a semi-permanent end use.

Parallel to the land-use change that has occurred is the urbanisation of rural land surrounding major urban centres. As the population grew, more people moved to and lived in cities and towns that had less and less interaction with farming activities. The growing urban–rural divide has brought contrasting perspectives about the natural environments that people live in and the expectations that are held on the desired state of such environments, all of which are driven by different value systems within our society.

**Societal impacts on farm-level decisions.** Our purpose in highlighting the above issues relating to pastoral-sourced pollution, methane emissions, and land-use change within New Zealand is not in any way to advocate for specific policies. Nor is it to imply that these are the only pastoral externalities of importance. Rather, it is to provide examples that highlight the effects of broad societal perspectives on farm and ranch-level decisions, with these perspectives typically mediated through government policies and regulations. These broad societal perspectives stem from all walks of life within the New Zealand society, ranging from urban to rural, and from indigenous to migrant populations who have varied historic connections to the land and the broader natural environments, and are, at times, shaped by the media from both within New Zealand and internationally.

Given this situation, transdisciplinary thinking becomes fundamental in relation to identifying stakeholders together with their value-based perspectives and, thereby, provides a framework in which a civil analysis appropriate to a democratic society can occur. Indeed, part of the rationale for this paper is a recognition by the authors that this framework is typically missing from debates about pastoralism both within our own and other countries. We also emphasise here that the bio-economic pastoral systems of New Zealand are socially constructed, with key stakeholders in the system operating some distance geographically from the pastoral location, and with interests quite separate from pastoralism itself.

### 4.2. Applying Transdisciplinary Thinking to the Pastoral Systems on the Qinghai-Tibet Plateau

At this point we change our focus from New Zealand to the Qinghai-Tibet Plateau (the Plateau) in the Qinghai Province in Western China, where two of us have had the privilege of working. In so doing, our purpose is to demonstrate that, although the principles of transdisciplinary thinking can be generalised, the outcomes of such thinking are very much a function of the context to which the principles are applied.

#### 4.2.1. Contextual Background of Henan County

This second case study will draw learning from some research that we have done in Henan County, Huangnan Prefecture of Qinghai Province, located on the east side of Qinghai-Tibet Plateau in Western China. We have had experiences visiting Henan County over the past decade. What we report here is therefore a collection of knowledge and insights from the literature, local knowledge, as well as our own observations and experiences. Contrasting to the less than 200 years of pastoral farming history in New Zealand, up on the Qinghai-Tibet Plateau pastoral farming has been the way of life for millennia, albeit significant changes in recent decades. Up until around the 1950s, nomadic herders lived on the Plateau, farming yaks and Tibetan sheep, which were almost entirely their sources of livelihood [40].

**Figure 2:**
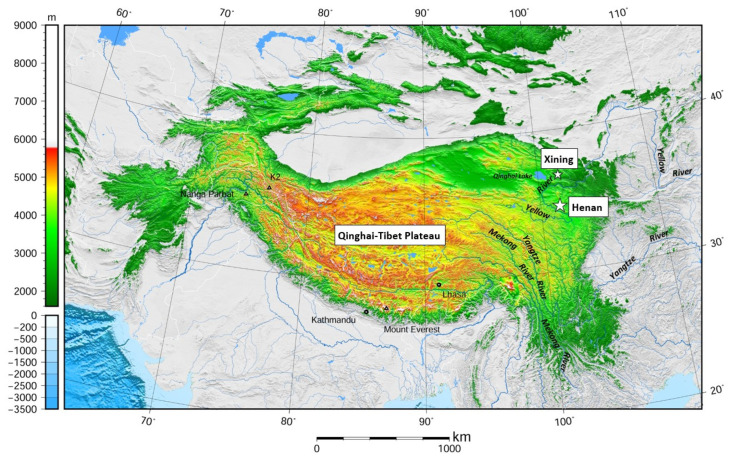
Map of Qinghai-Tibet Plateau [43] (used with permission).

Being the largest plateau on earth, the Qinghai-Tibet Plateau covers an area of 2.6 million square kilometres, with almost its entirety lying within the boundaries of China. The Plateau takes up more than a quarter of the total land area of China, spanning 31 degrees in longitude and nearly 25 degrees in latitude [41]. Grassland, deserts, and high-altitude mountains make up the main landscape of the Plateau, with much of it being above the tree line. There are in total approximately 1.7 million square kilometres of grassland on the Plateau, which has been the traditional home for Tibetan nomadic herders for millennia [40,42]. The significance of the Plateau goes beyond its sheer size. The northeastern part of the Plateau, which sits within Qinghai Province, is where three major rivers of China (the Yellow, Yangtze, and Mekong (known as Lancang in China)) all arise. The perceived environmental significance of this area by the Chinese Government subsequently led to the establishment of the Sanjiangyuan (三江源, Three-River Headwaters) National Nature Reserve, or Sanjiangyuan Region, which covers an area of 302,000 square kilometres and almost the entire southern half of Qinghai Province. Being an important nature reserve, the Chinese Government has decreed that all production systems within this reserve must be organic, and no chemical fertilisers are permitted for use. Such a decree puts limitations on what farming activities are allowed for the livestock farmers living within the region. The research site, Henan County, where much of the insights shared in this paper come from, is located in the eastern side of the Sanjiangyuan Region (Figure 2).

Henan County covers an area of 6700 square kilometres. Annual rainfall is 600 mm and is summer-dominant. We observed that pastures lay dormant until early May each year, primarily due to low soil temperatures and the exhaustion of moisture at the start of spring. The population of Henan County increased from 25,644 in 1991 to 39,508 in 2014 (1.9 percent compound growth per annum); during this time the farming population increased from 21,868 to 32,977 (1.8 percent compound growth per annum) [44,45].

Like the rest of the Plateau, the herders in Henan County maintained a nomadic farming and lifestyle since the beginning of time. Being above the tree line, the farming system has always been pastoral, with yaks and Tibetan sheep grazing on native pastures. Before the 1950s, groups of herders, which broadly reflected the communal structure, grazed the land through a seasonal rotation system. Herders moved their housing (mostly in tents) and livestock on the communally allocated grazing land according to the seasons. Miller [40] reported a tradition of herders undertaking a stocktake of livestock every three years, after which those whose herds had grown would get allocated more land and those whose herds shrunk would lose grazing land. The dominant religious belief on the Plateau has been Buddhism over the recent centuries, which continues to govern and direct their way of life.

This traditional nomadic farming system has been through significant changes since the 1950s after the Communist Party took over the reign of the Plateau from traditional Buddhist leaders. There has been a gradual transition towards sedentarisation of the herders, and most farming families now graze their livestock on fixed parcels of land. There have been fences built up to define the land boundaries between families, although the maintenance of these fences has not always been kept up in recent decades [43]. Within their own boundaries, some families have internal fences to separate the land up to four blocks and operate under a form of seasonal rotational grazing. Some families have two parcels of land, one for the summer and one for the winter, normally at a distance of within a day’s travel from each other. There are also some groups of farmers that still retain a form of communal grazing system. For example, we observed a farming family working within a group of 23 families, who shared the land and managed their livestock together in their grazing rotations [46]. Farmers’ livelihoods are dependent on the income from livestock products, mainly meat, milk, and yak dung, which is further processed into organic fertiliser. There are also retired farmers being relocated into the county townships, where their grandchildren go to boarding school and live with them on weekends during school terms [43]. This relocation of farmers, normally organised by the local government, is considered a way to reduce livelihood pressure on the grassland [47].

Much of the above-mentioned land-use policy change towards sedentarisation has been driven by the belief in “the tragedy of the common”, where communal land is vulnerable to overgrazing [48]. Whether or not fixing farmers to the land has helped to mitigate grassland degradation is something for another discussion. The reality of the grassland in Henan County, and much of the Plateau, is that degradation prevails. Indeed, through our own observations and communication with the local herders, the greatest challenge that is currently facing herders in Henan County, which is representative of many others on the Plateau, is ecological degradation. Older farmers lament at the fact that the pastures are no longer as tall as they remember them from their childhood. Rodent and caterpillar infestations (e.g., plateau pika *Ochotona curzoniae* [49], Tibetan dwarf hamsters *Cricetulus kamensis* [50], marmots *Marmota himalayana* [51], and Lepidoptera: Lymantriinae: *Gynaephora* [52]) are problematic for many herders because they consume large amounts of the biomass of the pastures (above and below ground), and the productivity of the grassland is declining.

This ecological degradation of the grassland presents itself as a wicked problem, in that finding a restoration solution is a complex challenge. For Henan County, being located within the Sanjiangyuan Region limits its production systems to be organic, so replenishing nutrients to the land through chemical fertilisers is not an option. Locals also argue that operating under the organic production system offers their ability to deliver niche products and demand premium prices on the markets, many of which are located well beyond the boundaries of the Plateau. Yet, with the depletion of nutrients through their removal from livestock products for human consumption as well as dung as fertilisers, with or without overgrazing, the productivity of the land inevitably continues to decline. With limited understanding of the soil biota and their interactions with a vast variety of native plant species within the native pastures on the Plateau, scientists are still searching for effective solutions to deter the degradation of the land.

Additionally, the dominance of Buddhist beliefs remains strong, in that farmers themselves would only ever slaughter the odd animals for home consumption, as Buddhism promotes preservation of life and no killing. Farmers also keep livestock dedicated to the Buddhas as redemptions for family members who fall ill. These animals often wear prayer bells and will remain on the farm until they die of old age. The livestock grown for the markets are often sold to Muslim operators who own the slaughterhouses. Farmers, therefore, are normally price-takers as they see no other outlets for themselves to do the slaughtering and processing. At the time of our research taking place, the institutional set-up of the livestock product value chain, particularly for yak meat, was somewhat less sophisticated. Animals were sold off the farm on a per-head basis without weighing, incentivising farmers to focus on increasing the number of livestock on the farm rather than their production performance [43].

Through a transdisciplinary and system perspective, we attempted to first find an explanation for the degradation of pastures, before a solution could be presented. Given the decree on organic production, we recommended the introduction of edible legumes to the Plateau to replenish key nutrients, such as nitrogen, into the system (after some preliminary soil test results indicating a lack of nitrogen) [43]. Our own research trialled a number of legume species with mixed success. While this was only the very first step of our attempt to help restore the grassland, we remain hopeful that improvements can be made. Nevertheless, much more work is yet to be done to make the change more sustainable. The introduced legume species need to be integrated into the farming systems that local herders can adopt and master, while still respecting their traditions and beliefs that have been passed down for centuries. Such an integration also needs to deliver products that can bring attractive returns to the farmers, so that there is an incentive to sustain such improved farming systems, which can ultimately lead to improved ecological balance on the Plateau.

#### 4.2.2. Stakeholders and Their Perspectives on the Pastoral Systems in Henan County

As we moved to identifying relevant stakeholders of the pastoral systems in Henan County, there were and are again some realities to be acknowledged, just as we did when analysing the New Zealand case. In the case of Henan County as a representation of a wider context of the Qinghai-Tibet Plateau, two fundamental realities are worth noting. Firstly, the communities living in Henan County, as well as those on the rest of the Plateau have, over the past century, have transitioned from a world of isolation (due to access barriers created through considerable land elevation) and, therefore, a closed system, to an open system where both human and agricultural products flow in and out of the system. The second reality to note is the significant influence of the Government, both central and local, on the institutions and infrastructure that the pastoral systems on the Plateau operate within. Much of this influence is for the purpose of improving livelihoods on the Plateau, as well as minimising the negative externalities of pastoral activities on the Plateau and further downstream of the big river systems.

**Grassland degradation.** One key consequence of the open system that the Plateau now operates under is the vast depletion of nutrients. Here we defined the boundary of the pastoral system on the Plateau as the physical boundaries of the Plateau. When it was operating under a closed system centuries ago, due to its inaccessibility brought by considerable elevation, the key elements within the system (e.g., nitrogen, phosphorus, and sulphur) cycled through human and animals but always returned to the system. This changed completely when modern roads and railways enabled the flow of people and products in and out of the Plateau. Pastoral products, such as meat and milk, are considered of premium quality and are in high demand from consumers living outside the Plateau, with some as far as Beijing and Shanghai in eastern China. Together with inadequate amount of nutrients replenishing the land, a major consequence of grazing intensification to meet such consumer demands is the ongoing export and consequent depletion of nutrients off the Plateau, causing widespread degradation of the grassland. For example, in our own research at Henan County, the grazing pressure on the grasslands of Henan appeared to have increased by a factor of approximately 2.8 between 1960 and 2015 [43]. To date, there has been limited carrying capacity modelling done in the context of Qinghai-Tibet Plateau. This is possibly due to the complex rotational grazing practised on the Plateau that has been evolved from a nomadic tradition, as well as the fact that many wild animals on the Plateau also graze the same land, adding complications to carry capacity calculation and modelling and, consequently, challenging to be used to guide degradation mitigation. Such degradation has a direct impact on the livelihoods of the farmers/herders on the Plateau, making them key stakeholders. Besides farmers, consumers of Plateau products and tourists who visit the Plateau are also stakeholders, as their consumption experiences are impacted by such degradation.

**Government intervention.** A significant stakeholder of the pastoral systems on the Qinghai-Tibet Plateau is the Government, both at the national and local levels. These government agencies direct and support farm activities on farms on behalf of the wider communities that live both on the Plateau as well as at lower altitudes, especially those within the catchments of the three major rivers within China. For example, the decree of organic farming only within the Sanjiangyuan Region is largely for the preservation of these important rivers systems at their critical source areas. As these government agencies have the wider community to answer to, and given their influence over decisions made both on and off farm at all levels under the political system that China operates within, they too are important stakeholders of the pastoral system on the Plateau.

**Societal impacts on farm-level decisions.** The societal impact on farm-level decisions as it plays out in the context of Qinghai-Tibet Plateau reflects an interaction of value systems. In behind the complex socio-ecological challenges present on the Plateau, as we observed through Henan County, is an interface where several beliefs and associated value systems collide. On the one hand, the presence of the Buddhist beliefs remains strong within the farming community, driving behaviours such as preserving livestock dedicated to Buddhas at the cost of farm productivity. On the other hand, the players along the value chain beyond the farm gate do not necessarily share the same beliefs, in that many processors have Muslim beliefs, and consumers from both on and off the Plateau are likely to have come from diverse backgrounds and, hence, different value systems, while nevertheless appreciating the credence attributes associated with the Plateau. The end result of such a value system collision is that livestock numbers increased at a speed that changed the ecological balance of the Plateau, leading to widespread degradation.

Consequently, in applying transdisciplinary thinking to search for solutions to the challenges faced by the Plateau, there needs to be a recognition of the different value systems at play that are linked to a wide spectrum of stakeholders living both on and off the Plateau. Whatever solution to be proposed, therefore, needs to strike a balance between the benefits to these stakeholders in recognition of their diverse perspectives.

## 5. Discussion

The two cases presented here both have striking contrasts and common elements. The contrasts derive from the different contexts, with stark differences in scale, biophysical resources, human resources, pastoral history, and political systems. The commonalities derive from the attempt to identify and understand all stakeholders, with this leading to a focus on externalities beyond the pastoral system itself.

Despite operating within very different political systems, in each case it is stakeholders beyond the narrowly defined pastoral location who have determined the regulatory framework within which pastoral livelihoods are conducted. We suggest that this regulatory framework is, in both cases, a relatively modern imposition imposed by broader society and deriving from increased awareness at a national level of the externalities. Both cases presented some confronting perspectives of the relevant stakeholders, in that satisfying the wishes of one perspective would almost certainly come at the cost of sacrifices made by another. The transdisciplinary thinking will, therefore, serve the purpose of creating the pathway of understanding these contrasting perspectives and provide the basis for potentially balanced solutions for the wicked problems at hand.

We emphasised earlier in this paper that transdisciplinary thinking always starts with a context and that is how each of our two case studies here have been presented. The geographical location of the physical system may be either broad or restricted, and the two cases illustrate this, with one taking a country perspective and the other constrained to a specific county. We contend that, regardless of the chosen geographical boundaries, a genuine transdisciplinary analytical process that is undertaken from a human activity system perspective leads to the acknowledgement that the overarching system is Planet Earth. Lesser geographical systems are essentially sub-systems chosen for reasons of practicality of analysis and subsequent actions. A transdisciplinary approach requires acknowledgement that externalities extend beyond the boundaries by planetary sub-systems.

Earlier in this paper, we highlighted the difference between transdisciplinary thinking and transdisciplinary actions. We acknowledge here that transdisciplinary thinking when applied to problem identification does not, by itself, necessarily provide a pathway leading all the way to transdisciplinary system solutions or actions that are acceptable to all stakeholders. This is because the nature of a wicked problem is that there may be no solution that all parties consider acceptable.

What we do contend, however, is that transdisciplinary thinking about pastoralism provides a pathway to the comprehensive wide-ranging identification of relevant issues and perspectives. It starts by identifying a context and recognising that transdisciplinary thinking is always about human activity systems. This recognition facilitates contextual structuring that gives acknowledgement to diverse objectives, thus avoiding implicit but unstated judgements about which objectives and values might take precedence. Author reflexivity is an internal analytical process that can help avoid these same unstated judgments.

In our discussions that focused on the transdisciplinary thinking at the system level, we recognise that there have been inevitably omissions of some important elements within these systems. For example, the indigenous perspectives and gender perspectives within these systems are deeply intertwined with the local history and cultural norms. In New Zealand, how the indigenous (Māori) people employ transdisciplinary thinking in search of potential solutions for the wicked problems related to pastoral farming are guided by their connection to the land in both the physical and the spiritual sense [53]. In parallel, defined gender roles within pastoral farming on the Qinghai-Tibet Plateau remain firmly engrained in people’s livelihoods, despite the changes that have occurred over the last century [43]. These indigenous and/or gender perspectives are complex matters in their own right, and hence deserve dedicated scholarly attention in the future.

## 6. Conclusions

Reflecting on the complex wicked problems that each of the case studies present, we suggest that many of the controversies surrounding appropriate strategies for pastoral systems are a consequence of a failure to identify and acknowledge the full range of stakeholders and associated value systems. At the very least, transdisciplinary thinking provides a framework for civilised debate and communication within a broader framework of policy generation. In this regard, transdisciplinary thinking is not in competition with disciplinary, multidisciplinary, or interdisciplinary paradigms, but is a fundamental organisational tool for structuring complex human activity systems and issues within a constrained global environment. Even if specific actions are focused on specific system components, there is a benefit of assessing pastoral policies within a transdisciplinary framework.

## Data Availability

No new data were created or analysed in this study. Data sharing is not applicable to this article.
